# Diagnostic delay in cerebral creatine deficiency disorders: lessons learned from a cross-sectional single center study, and guanidinoacetate and creatine measurements in Switzerland between 2015 and 2023

**DOI:** 10.1186/s40348-024-00188-4

**Published:** 2025-01-22

**Authors:** Christina Kaufman, Anaïs D’Andrea, Annette Hackenberg, Martin Poms, Olivier Braissant, Johannes Häberle

**Affiliations:** 1 Division of Metabolism and Children`s Research Center, University Children’s Hospital Zurich, Lenggstr. 30, 8008 Zurich, Switzerland; 2 Division of Pediatric Neurology, University Children’s Hospital Zurich, Zurich, Switzerland; 3 Division of Clinical Chemistry and Biochemistry, University Children’s Hospital Zurich, Zurich, Switzerland; 4 Service of Clinical Chemistry, University of Lausanne and Lausanne University Hospital, Lausanne, Switzerland

**Keywords:** Cerebral creatine deficiency, Diagnostic delay, Expressive speech delay, Global developmental delay, Cognitive impairment, Substitution therapy

## Abstract

**Background:**

Cerebral creatine deficiency disorders (CCDD) are rare diseases caused by defects in the enzymes L-arginine: glycine amidinotransferase (AGAT) or guanidinoacetate-N-methyltransferase (GAMT), which are involved in synthesis of creatine; or by a defect in the creatine transporter (CRTR), which is essential for uptake of creatine as important energy source into the target cells. Patients with CCDD can present with a variety of unspecific symptoms: global developmental delay, speech-language disorder, behavioral abnormalities and seizures. Early treatment initiation is essential in AGAT and GAMT deficiencies to achieve a favorable outcome. This study describes the CCDD patient cohort in a single center, and an analysis of the referrals to two Swiss laboratories in Lausanne and Zurich between 2015 and 2023 for the two marker metabolites guanidinoacetate and creatine.

**Results:**

The patient cohort comprised 6 patients (defects: 2 GAMT, 4 CRTR), who were initially seen by different subspecialties depending on first symptoms. There was a diagnostic and therapeutic delay between 3 and 32 months (mean 13.8). Numbers of biomarker requests showed a constant increase during the study period, with a majority of tests performed in urine, the preferred sample for CCDD detection. Almost all samples (93.3%) were sent in by large hospitals (mainly from neurology, developmental pediatrics and metabolism) and only few (5.2%) by pediatricians in private practice, although those usually see the patients first.

**Conclusions:**

The data from this study demonstrate a relevant delay in identifying patients with these rare conditions, and a predominance of biomarker analysis requested from pediatric subspecialties that are involved in patient management often long after occurrence of symptoms. To reduce the diagnostic delay and the outcome of patients, the current practice of sample referral should be reflected and first-contact healthcare providers should be encouraged to initiate selective screening.

**Supplementary Information:**

The online version contains supplementary material available at 10.1186/s40348-024-00188-4.

## Background

Cerebral creatine deficiency disorders (CCDD) are a group of rare diseases comprising L-arginine: glycine amidinotransferase (AGAT) deficiency (OMIM #612718), guanidinoacetate-N-methyltransferase (GAMT) deficiency (OMIM #612736) and creatine transporter (CRTR) deficiency (OMIM #300352) [16]. All three defects result in reduced creatine availability in target tissues, especially in muscle and brain [22]. Creatine is a semi-essential compound, which is used in tissues with high energy demand to generate a phosphocreatine pool for rapid adenosine triphosphate (ATP) regeneration.

**Fig. 1:**
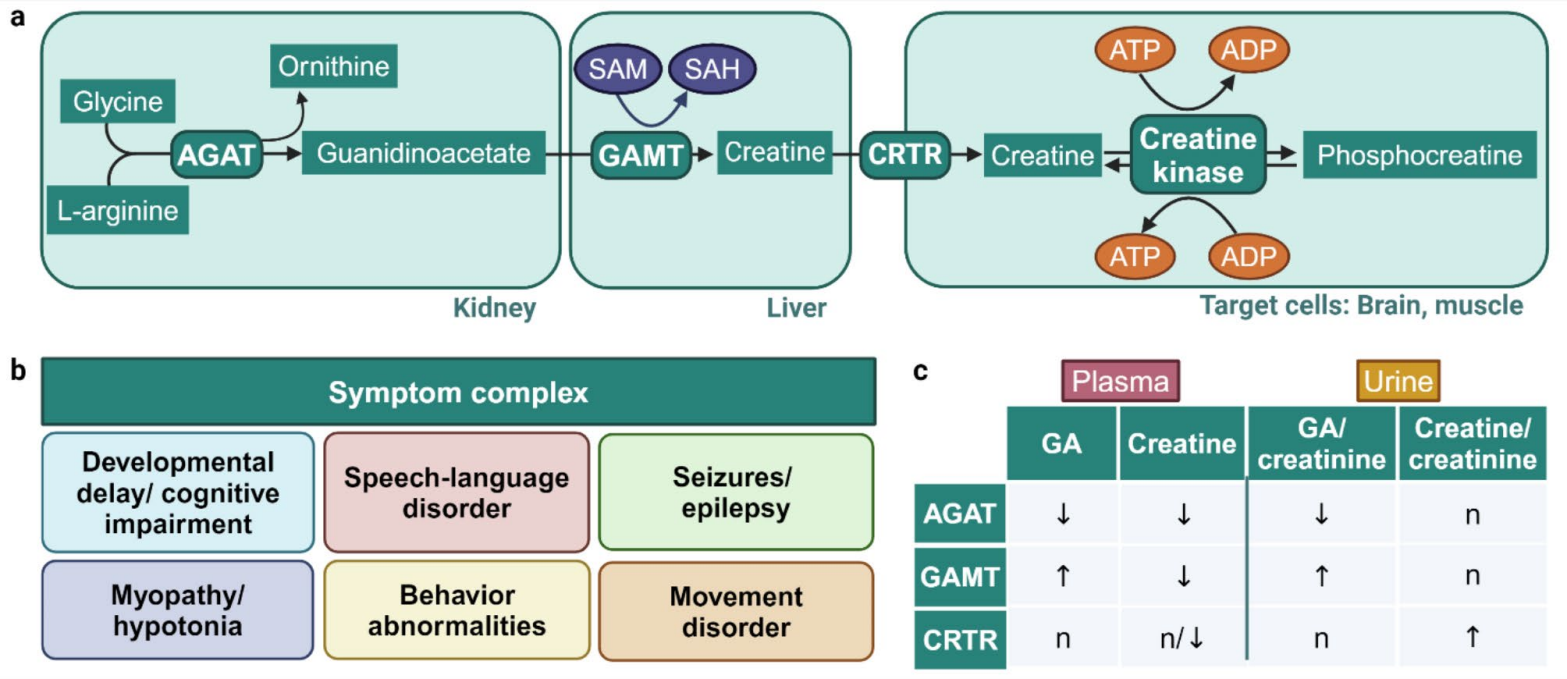
Creatine biosynthesis pathway, CCDD symptom complex and diagnostics

Creatine is only partially taken up from dietary sources (about 50% in a non-vegetarian). About 50% are synthesized in a two step process: AGAT (mainly expressed in kidneys) uses the two substrates glycine and arginine to form guanidinoacetate (GA), which is methylated by GAMT to form creatine [21, 28, 30]. This second step by GAMT uses S-adenosylmethionine (SAM) as methyl donor and mainly takes place in the liver [28] (Fig. 1a). Creatine is finally taken up into the target cells via CRTR, where it is used as substrate by the tissue specific creatine kinase to produce phosphocreatine. Phosphocreatine serves as an energy storage allowing rapid replenishment of ATP in tissues with high energy requirements, such as the skeletal muscle, myocardium and brain [30]. Besides its role for ATP regeneration, creatine is an important osmolyte in the brain, and probably a neuromodulator or even true neurotransmitter [2]. Due to the poor permeability of the blood-brain barrier (BBB) for creatine, the brain has to have the machinery to synthesize creatine, hence AGAT and GAMT are also expressed in this organ [4]. Creatine pools must be constantly refilled since creatine spontaneously converts to creatinine, which passively diffuses through cell membranes and is excreted via the kidneys [29].

The three CCDDs share common clinical features including global developmental delay, cognitive impairment, speech-language disorder, behavior problems, muscular hypotonia and seizures [10, 23, 24, 26] (Fig. 1b). Seizures and epilepsy seem to be more specific for GAMT and CRTR defects [23, 26], whereas muscle weakness or myopathy seem to be more typical for AGAT deficiency [10].

For diagnostic purposes, the metabolites GA and creatine can be measured in blood, urine and cerebrospinal fluid using liquid chromatography–tandem mass spectrometry (LC-MS/MS) or, probably less sensitive, gas chromatography–mass spectrometry (GC-MS) [19]. Usually analyses in blood and/or urine are performed, but urine is the only material allowing reliable detection of all three defects while patients with a CRTR defect may be missed in plasma (Fig. 1c). After positive biochemical testing, the diagnosis should be confirmed by brain magnetic resonance spectroscopy (MRS) measuring creatine and phosphocreatine peaks and/or genetics [6]. Newborn screening for GAMT deficiency has been implemented in several countries based on GA determination in dried blood spots [17, 20].

AGAT and GAMT deficiencies are caused by bi-allelic sequence variants in *GATM* and *GAMT*, respectively. CRTR is encoded by *SLC6A8*, which is located on the X-chromosome. In the case of an unfavorable X-inactivation pattern, female heterozygotes may present with mild symptoms such as mental retardation and learning disabilities [25].

AGAT and GAMT deficiency are treated with oral creatine supplementation. To reduce the production of the neurotoxic GA, patients with GAMT deficiency should be supplemented with high dose L-ornithine and an arginine-restricted diet should be considered [23]. In AGAT and GAMT deficiencies, treatment initiation before 9 months of age can result in (near) normal development [23]. Treatment of CRTR deficiency with creatine and the precursors arginine and glycine is far less successful and so far, no efficient treatment is available for this condition: neither creatine, nor the creatine precursors arginine and glycine, nor creatine derivatives (many different have been tried, including cyclocreatine) have shown sufficient efficiency. The only exception are few females with CRTR deficiency with a favorable X-inactivation especially probably at the BBB, who can benefit from creatine supplementation [7, 9]. Betaine and SAM have been explored in the past without showing clear treatment benefit [13, 18].

To ensure an early start of treatment in CCDD patients amenable to therapy, early diagnosis, ideally already in infancy, would be essential. However, several studies suggest that CCDDs are underdiagnosed: a prospective single center study including subjects with mild to severe mental retardation showed a CCDDs prevalence of 2.7% [15]; a retrospective multicenter study with more than 6000 patients with unexplained neurological symptoms found a CCDD prevalence of 0.25%. In this latter study, diagnosis of GAMT or CRTR defects was established at a median age of 5 years (range: 2–29) or 9.5 years (range: 2.5–28), respectively [5].

Aims of the present study were to investigate in a cross-sectional effort whether also the patients treated in our center had suffered a diagnostic delay, and to evaluate the situation of the selective screening of CCDDs targeting the marker metabolites for these conditions since introduction of an LC-MS/MS method. Hereby, we found a relevant diagnostic delay in our small patient cohort, which is probably at least partially caused by a long patient journey and a corresponding delay in performing appropriate selective metabolic screening.

## Methods

### Aim, design and setting of study

Aim of the present study was to investigate the diagnostic delay in a single center cohort (University Children’s Hospital Zurich). Focus was also on the institution and subspecialty that initially received patient referrals. Additionally, the GA and creatine measurements, as biomarkers for CCDD, were analyzed for the period between 2015 and 2023 in the two only laboratories in Switzerland, in Lausanne and Zurich, providing this biomarker assessment. Focus here was on the evolution of the number of analyses during the study period and whether analysis was requested in urine or plasma.

### Patient cohort

Data from all CCDD patients treated at the University Children’s Hospital Zurich between January 2015 and December 2023 were collected. The diagnosis was confirmed by molecular genetic testing and brain MRS in all patients. Patient files were reviewed and an item list containing information on age, sex, clinical signs and symptoms at diagnosis, patient and family history, anthropometric data, developmental assessments [1, 3, 8, 11, 14, 27], laboratory data including genetic findings, brain imaging including MRS, results of electroencephalogram (EEG), and treatment regimens were collected (details in Suppl. Mat.).

Diagnostic delay was determined as time between first referral to any subspecialty at the University Children’s Hospital Zurich and confirmation of the diagnosis by either molecular genetic testing or brain MRS.

### Analysis of biomarkers (guanidinoacetate and creatine)

GA and creatine measurements performed between January 2015 and October 2023 (Lausanne, Centre Hospitalier Universitaire Vaudois, CHUV) or December 2023 (Zurich, University Children’s Hospital Zurich) were extracted from the respective laboratory data bases and analyzed in regard to sample type (blood/ urine) and referring institution (hospital, private practice, other laboratory). Since the two laboratories in Lausanne and Zurich are the only providers of GA/creatine measurements in Switzerland, this presents a dataset for the entire country. Both laboratories use an accredited LC-MS/MS method since early 2015. Repetitive measurements for the same patients were deleted, and only the first measurements included in the analysis. To obtain a dataset comparable between both centers, for the year 2023 the total number of measurements was extrapolated for the missing months from the Lausanne laboratory (November/December) by calculating the average sample number per day for the period of January to October 2023 and adding the average of two months to the number of performed measurements. For analysis of referred sample type (urine, plasma or both), we considered urine and plasma samples as “pairs” if samples were received within three months for the same patient, since it is common practice to receive urine samples by mail from the families shortly after the patient was seen at the outpatient clinic.

To obtain details on the exact origin of the selective screening sample, we analyzed for the year 2022 the referring subspecialties from within the University Children’s Hospital Zurich.

## Results

### Patient cohort

At the University Children’s Hospital Zurich, a total of six patients (1 female, 5 male) aged 2.3 to 20.8 years old were treated during the study period from 2015 to 2023. Two patients were diagnosed with a GAMT deficiency (both of consanguineous descent and with Arab and Caucasian ethnicity from Syria and Albania, respectively) and four with a CRTR deficiency (three Caucasian ethnicity from Balkan region and Switzerland, and one Ashkenazi from Israel). Diagnosis was initially based on laboratory data from the selective metabolic screening but later confirmed in all patients by genetic means (with pathogenicity of identified sequence variants predicted if they were nonsense or splice variants and/or predicted in silico; details in Suppl. Table). Key biochemical findings were elevated guanidinoacetate in urine in the two GAMT deficiency patients (803 and 1912 mmol/mol creatinine, reference 15–152) and increased urinary creatine in the four CRTR patients (5280, 4900, 3110, and 4890 mmol/mol creatinine, respectively, reference 19-1046). The most common clinical features were lack of expressive speech (6/6), severe developmental delay (EQ < 50 in 5/6), movement disorders (2/6) and epilepsy (3/6). First symptoms were developmental delay (6/6) and febrile seizures (2/6) diagnosed between the age of 6 and 20 months. Patients were referred to our center at a mean age of 4.1 years (range: 1.6–11.1) and the mean age at diagnosis was 5.2 (range: 2- 13.8). The mean diagnostic delay was 13.8 months (range: 3–32). Half of the patients (3/6 patients) were initially referred to neurology due to epilepsy and/or severe global developmental delay, two patients to speech therapy and the ear-nose-throat (ENT) department due to severely delayed expressive language and one patient to the emergency department due to a febrile seizure. Details to the patient cohort are shown in Table 1.

**Table 1:** Patient cohort CRTR: creatine transporter; ENT: ear nose throat; GAMT: guanidinoacetate-N-methyltransferase; F: female; M: male

Patient	Defect of	Age at last visit (years)	Sex	Leading symptoms	First symptoms	Age at first symptoms (months)	Age at diagnosis (years)	Age at first referral (years)/ department	Diagnostic delay (months)	Development quotient < 50	Delayed speech	Epilepsy / seizures	Movement disorders	Gene: genotype
1	GAMT	20 10/12	M	Epilepsy, global developmental delay including speech	Severe developmental delay	10	13 9/12	11 1/12 neurology	32	yes	yes	yes	no	*GAMT*: c.64dupG, p.Ala22Glyfs*63 homozygous
2	GAMT	9 6/12	F	Epilepsy, no speech development	Febrile seizures, developmental delay	18	6 3/12	6 0/12 neurology	3	yes	yes	yes	no	*GAMT*: c.391 + 5G > C, p? homozygous
3	CRTR	8 10/12	M	Severely delayed expressive language, movement disorder	Speech and developmental delay	15	3 7/12	2 2/12 speech therapy	17	yes	yes	no	yes	*SLC6A8*: c.1133 C > A, p.Ala378Glu hemizygous
4	CRTR	3 10/12	M	Delayed expressive language development	Speech delay	20	2 4/12	1 10/12 ENT	6	Not investigated	yes	yes	no	*SLC6A8*: c.11474_1484del, p.Val492Leufs*6 hemizygous
5	CRTR	2 4/12	M	Global developmental delay including speech	Febrile seizure with status epilepticus, developmental delay	12	2 0/12	1 7/12 emergency department	5	yes	yes	no	no	*SLC6A8*: c.190T > C;1674G > C, p.Ser64Pro; Glu558Asp hemizygous
6	CRTR	8 7/12	M	Development and speech delay, hypotonia, atactic movement disorder	Muscle hypotonia, developmental delay	6	3 6/12	1 10/12 neurology	20	yes	yes	no	yes	*SLC6A8*: c.528G > C, p.Trp176Cys hemizygous

CRTR: creatine transporter; ENT: ear nose throat; GAMT: guanidinoacetate-N-methyltransferase; F: female; M: male

### Biomarker analysis in Switzerland between 2015 and 2023

**Fig. 2:**
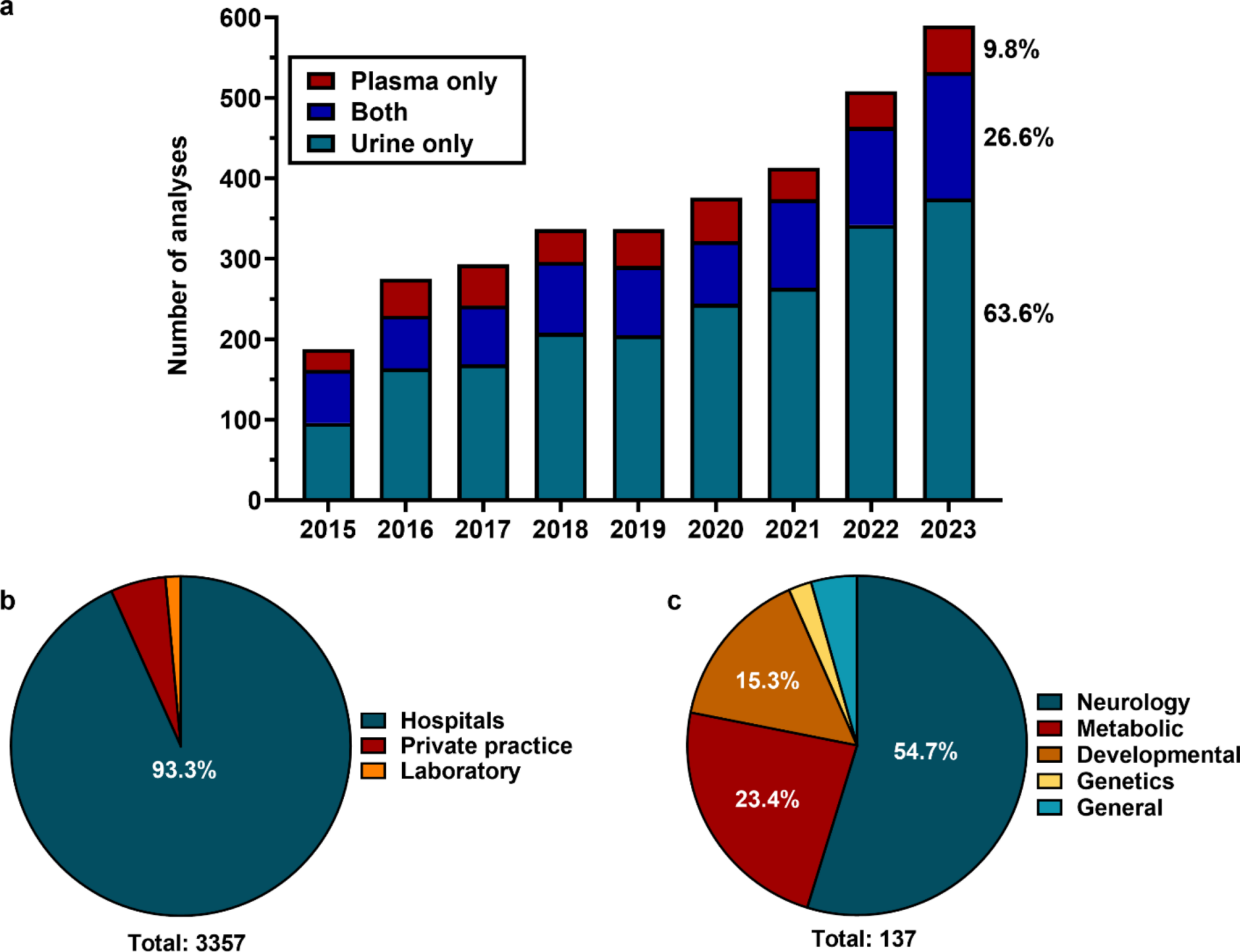
GA and creatine measurements in Switzerland between 2015 and 2023 (**a**) Number of GA and creatine analyses performed between 2015 and 2023 in the Zurich and Lausanne laboratories. (**b**) Origin of referred samples (hospital, private practice, laboratory) for the period between 2015 and 2023. (c) Overview of referring specialty within the University Children’s Hospital Zurich in 2022

During the selected period (January 2015 to December 2023), a total of 4967 GA and creatine measurements were performed in urine and plasma, 3335 analyses in Zurich and 1632 in Lausanne (including repetitive measurements for the same patient) (Fig. 2a). At initial referral, analysis was requested in both urine and plasma in 845 cases, in 2067 cases only urine was analyzed and in 405 cases only plasma was analyzed. There was only a small change in the proportion of samples between 2015 and 2023: urine was sent in an average of 61.2% (range: 51.1–67.3) of cases in each of the respective years, both urine and plasma in an average of 25.9% (range: 20.7–35.1) and plasma in 12.9% (range: 8.7–17.4) of cases. Most of the samples were sent by hospitals (93.3%), and only few from private practices (5.2%) and private laboratories (1.5%) (Fig. 2b). Tertiary hospitals sent 79.9% of samples and 13.4% were sent by other hospitals.

The number of analyzed samples steadily increased in the study period with 312 samples measured in 2015 and 883 measured in 2023 (Fig. 2a). The proportion of analyses using only plasma samples has decreased from 15.3% in 2015 to 6% in 2023. In 2022, most samples were sent from neurology (54.7%), metabolism (23.4%), developmental pediatrics (15.3%), and only few from medical genetics (2.2%) and general pediatrics (4.4%) (including a single request from the ENT department) (Fig. 2c).

## Discussion

Disorders of creatine biosynthesis belong, with the current exception of most patients with CRTR defects, in principle to the treatable inborn errors of metabolism. However, treatability and outcome of patients largely depend on an early diagnosis. With the introduction of highly precise diagnostic tools into the selective screening, namely the LC-MS/MS methodology, all should be set for identifying patients early after onset of first symptoms. However, based on reports from literature and our own experience, this timely diagnosis of CCDD patients remains a challenge. We were interested to study in detail the situation in our current patient cohort, and to evaluate the respective selective screening done in Switzerland.

To achieve these aims, we first analyzed a single center CCDD patient cohort consisting of six patients **(**2 GAMT and 4 CRTR patients) with an emphasis on symptoms at first contact, involved first contact specialties, symptoms at the time of referral and the diagnostic delay seen in these patients. In a second part, all GA and creatine measurements performed in Switzerland over a period of eight years were looked at in detail to learn more about which type of samples were analyzed (urine/ plasma) and who exactly ordered CCDD selective screening.

Depending on their leading symptoms, patients in our cohort were referred to different departments within the University Children’s Hospital Zurich. Patients with epilepsy and severe global developmental delay were first seen by pediatric neurologists; a patient with a complicated febrile seizure was initially seen in the emergency department; two patients with a severe expressive language delay were initially seen in speech therapy and the ENT department. Thus, due to the generally unspecific and variable symptoms of CCDD patients, the diagnostic path may strongly vary depending on dominating first symptoms. However, despite the involvement of specialists, the average diagnostic delay was still extensive (mean 13.8 months) with a likely negative impact on patient benefit from substitution therapy.

Regarding the laboratory evaluation, analysis of GA and creatine measurements showed a steady increase of analyses during the study period, and in 2023 almost 90% of referrals included the recommended sample type, urine (as “urine only” or “plasma and urine”) (Fig. 2a). Both these trends are encouraging, which is especially the case for the preferred sample type. Urine is clearly the best matrix for the initial selective screening and to detect all three disease entities (defects of AGAT, GAMT and CRTR) while the combined analysis of urine and plasma could be superior for follow-up of a suspicious urine sample based on better specificity in plasma. Interestingly, almost all samples (> 90%) were referred by large hospitals and only around 5% by pediatricians in private practice. In fact, in 2022 only 16 paediatricians out of a total of 1366 paediatricians in private practice in Switzerland (https://www.fmh.ch/themen/aerztestatistik/fmh-aerztestatistik.cfm) have sent any sample (a total of 25 samples). This pattern has not changed over the past eight years, suggesting that the increase in awareness for these diseases is mainly occurring at tertiary places. Within the University Children’s Hospital Zurich, > 90% of samples were sent in by neurology (54.7% of all referrals), metabolism and developmental pediatrics, which is in line with a previous study reporting the largest proportion of diagnoses made by neuropediatricians [5].

With respect to improving the diagnostic situation for CCDD patients, it is of note to realize that in our study, first contact specialties belonged to departments with the lowest number of screening requests. For example, the ENT department, which was the first contact department for one of our patients and which is likely the first contact for many other patients seeking advice for speech delay, had only referred a single urine sample in 2022 (total samples: 137). This may be due to lack of diagnostic guidelines in certain subspecialties; at least, it is interesting to note that neurology, metabolism and developmental pediatrics provide internal or official guidelines for the screening of CCDD, but may see patients only at a more advanced stage of their disease. Based on this, we suggest considering implementation of standard procedures (“red flags”) for patients with certain symptoms (for instance “severe speech delay at 2 years of age”) to initiate a selective screening for CCDD by means of GA and creatine analysis in urine. This may be especially effective if done by those healthcare providers that are likely first contact groups such as general pediatricians, ENT specialists, or speech therapists. An interesting development in the field is the recent introduction of newborn screening for GAMT deficiency in some of the US states, which may become key for presymptomatic identification of patients [12]; while the latter is part of a general screening using a biomarker for identification of affected patients, also early genetic diagnostics in children with neurological symptoms by whole-exome sequencing could result in a faster detection of CCDD patients.

One limitation of the study is its single center character resulting in only a small number of patients. As another limitation, we were only able to analyze the origin of the sample in detail for some of the institutions as many hospitals do not disclose the precise referring departments. As a result, the most detailed information was available from the University Children’s Hospital Zurich.

## Conclusions

In summary, our single center patient cohort showed a large diagnostic delay. This is unfortunate since early treatment initiation is essential for a favorable patient outcome. A common early symptom in our cohort was severe expressive speech delay, which would ideally trigger selective screening in first contact groups e.g. pediatricians in private practice, ENT specialists, or speech therapists. Also in our study, most analyses were requested by (large) hospitals and within those, most were requested by neuropediatricians. We conclude from these data, that strategies should be developed to identify CCDD patients earlier. Current diagnostic pathways should be reevaluated to implement selective screening for treatable metabolic diseases at an earlier time point.

## Electronic Supplementary Material

Below is the link to the electronic supplementary material.

Supplementary Material 1

Supplementary Material 2

## Data Availability

Data is provided within the manuscript and supplements. Raw data is available upon reasonable request from the corresponding author.

## Declarations

### Data presentation

Figures were created with BioRender.com. Graphs were designed using Graph Pad Prism (Version 10.1.2 for Windows, GraphPad Software, Boston, Massachusetts USA, www.graphpad.com).

## Abbreviations

AGAT: L-arginine: glycine amidinotransferase
ATP: Adenosine triphosphate
CCDD: Cerebral creatine deficiency disorders
CRTR: Creatine transporter
ENT: Ear, nose and throat medicine
GAMT: Guanidinoacetate-N-methyltransferase
GA: Guanidinoacetate
LC-MS/MS: Liquid chromatography–tandem mass spectrometry
GC-MS: Gas chromatography–mass spectrometry
MRS: Magnetic resonance spectroscopy
SAM: S-adenosylmethionine
SAH: S-adenosylhomocysteine
