# Supplementary material for: Diagnostic delay in cerebral creatine deficiency disorders: lessons learned from a cross-sectional single center study, and guanidinoacetate and creatine measurements in Switzerland between 2015 and 2023
Source: Mol Cell Pediatr. 2025 Jan 22;12:1. doi: 10.1186/s40348-024-00188-4 (PMC11751272; doi:10.1186/s40348-024-00188-4)
Supplement: Supplementary file 1 — Supplementary Material 1 [file 40348_2024_188_MOESM1_ESM.docx]

**Diagnostic delay in cerebral creatine deficiency disorders: Lessons learned from a cross-sectional single center study, and guanidinoacetate and creatine measurements in Switzerland between 2015 and 2023**

Christina Kaufman^1^, Anaïs D’Andrea^1^, Annette Hackenberg^2^, Martin Poms^3^, Olivier Braissant^4^, Johannes Häberle^1^

^1^Division of Metabolism and Children`s Research Center, University Children's Hospital Zurich

^2^Division of Pediatric Neurology, University Children's Hospital Zurich

^3^Division of Clinical Chemistry and Biochemistry, University Children's Hospital Zurich

^4^Service of Clinical Chemistry, University of Lausanne and Lausanne University Hospital, Lausanne

**Supplementary material**

1. ***Item list laboratory data***

- Date of laboratory analysis
- Sample type (urine/plasma)
- Institution (hospital, private practice, other laboratory) that submitted the sample

1. ***Top five referring institutions for guanidinoacetate and creatine measurements in Switzerland between 2015 and 2023***

| **Top five referring institutions for guanidinoacetate and creatine measurements in Switzerland between 2015 and 2023** | | |
| --- | --- | --- |
|  | *Number of analyses* | *%* |
| 1. University Children’s Hospital Zurich | 976 | 29.07% |
| 1. Lausanne University Hospital, CHUV | 554 | 16.50% |
| 1. Children's Hospital St. Gallen | 486 | 14.48% |
| 1. University Children’s Hospital Basel | 413 | 12.30% |
| 1. University Hospital of Bern | 208 | 6.20% |

1. ***Item list clinical data patient cohort University Children’s Hospital Zürich***

| **Biographical data** | Age | Sex | Diagnosis | Age at diagnosis | Age at first symptoms | Age at first referral/  Department | Diagnostic delay | Age at treatment start | Gene mutation |
| --- | --- | --- | --- | --- | --- | --- | --- | --- | --- |
| **Symptoms and clinical signs** | Speech (present yes/no) | Cognitive disorders (mild=0, moderate = 1, severe = 2) | Development age (at diagnosis /now) | Development quotient (at diagnosis/ now) | Movement disorders/ hypotonia (yes/no) | EEG abnormalities (yes/no) | Epilepsy / seizures (yes/no) | Now seizure-free (yes/no) | Autism spectrum disorder/ challenging behavior |
|  | Gait abnormality | Muscle hypertonia | First symptoms | Leading symptoms |  |  |  |  |  |
| **Personal history** | Term birth (yes/no) | Spontaneous delivery (yes/no) | Sit up (age in months) | Crawling (age in months) | Walking (age in months) | Microcephaly (yes/no) | Facial dysmorphism (yes/no) | Oligo-/anhydramnios | Traumatic brain injury (TBI) |
|  | Pes calcaneus | Pes planovalgus | Comorbidity | Complex febrile seizures |  |  |  |  |  |
| **Family history** | Other family members affected | Ethnic group | Habitual abortion | Consanguinity |  |  |  |  |  |
| **Anthropometric measurements (percentile)** | Height | Weight | Head circumference | BMI |  |  |  |  |  |
| **Laboratory data** | Urine creatine/ creatinine (mmol/mol, before and after treatment) | Urine guanidinoacetate/ creatinine (mmol/mol, before and after treatment) | Urine creatinine (mmol/L, before and after treatment) | Plasma creatine (μmol/L, before and after treatment) | Plasma guanidinoacetate (μmol/L, before and after treatment) | Liquor guanidinoacetate (μmol/L, before and after treatment) | Liquor creatine (μmol/L, before and after treatment) |  |  |
| **MRI/MRS findings** | Structural MRI abnormalities (yes/no) | Creatine peak basal ganglia (before and after treatment) | Creatine peak white matter (before and after treatment) |  |  |  |  |  |  |
| **Treatment** | Creatine supplementation dosis (mg/kg/day) | Ornithine dosis (mg/kg/day) | Glycine (mg/kg/day) | Arginine (mg/kg/day) | Currently on anti-seizure medication (yes/no) | Other medication (yes/no) | Speech therapy (yes/no) | Physiotherapy (yes/no) | Early education (yes/no) |
